# THE LAST MONTH OF LIFE: AN EXPLORATORY REVIEW OF THE NATIONAL HEALTH AND AGING TRENDS STUDY

**DOI:** 10.1093/geroni/igz038.1016

**Published:** 2019-11-08

**Authors:** Abigail Latimer, Lauren Montemuro-Rode, Brianna Garrison, Allison Gibson

**Affiliations:** 1 University of Kentucky College of Social Work, Lexington, Kentucky, United States; 2 Bryn Mawr College, Bryn Mawr, Pennsylvania, United States; 3 Baylor University, Houston, Texas, United States; 4 University of Kentucky, Lexington, Kentucky, United States

## Abstract

Approximately 80% of Americans prefer to die at home. Hospice and palliative care services are associated with improved pain and symptom management, increasing capacity to meet preferences for end-of-life care at home. However, according to the NHPCO (2018) only 48% of Medicare beneficiaries were enrolled in hospice at the time of death. This poster presents trends in the last month of life for adult Medicare beneficiaries age 65 or older examining the influential factors contributing to the quality of end-of-life experiences. A cross-sectional survey design was utilized with the National Health and Aging Trends Study (NHATS). Descriptive and inferential statistics were generated to describe a sample of persons (n= 241) who died in 2017. The sample demographics are predominately white (77.6%) females (61.4%) over 90 years old (42.4%). 29.5% of individuals died at home, 29.5% at the hospital, and 27% at a nursing home. Only 32.2% had hospice care in the last month, with many experiencing pain (71.1%), shortness of breath (54.7%), and anxiety/sadness (56.9%). There were 33.6% of participants who lived alone at death and 70% did not receive hospice care. The majority of these individuals were widowed (70.4%) and 33.3% died in the hospital. The other 28.4% died at their home or someone else’s and 25.9% died in a nursing home. Many older adults face multiple barriers to experiencing a quality end-of-life experience. Future research should examine the challenges facing those living alone at time of death.

